# Comparative Measurements of Local Seismic Rotations by Three Independent Methods

**DOI:** 10.3390/s20195679

**Published:** 2020-10-05

**Authors:** Johana Brokešová, Jiří Málek

**Affiliations:** 1 Department of Geophysics, Faculty of Mathematics and Physics, Charles University, V Holešovičkách 2, 180 00 Prague, Czech Republic; 2 Department of Seismotectonics, Institute of Rock Structure and Mechanics, Academy of Sciences of the Czech Republic, V Holešovičkách 41, 182 09 Prague, Czech Republic; malek@irsm.cas.cz

**Keywords:** seismic rotation, rotational seismometer, Rotaphone, seismic array

## Abstract

A comparative active experiment that is aimed at collocated measurement of seismic rotation rates along three orthogonal axes by means of three different methods is described. The rotation rates in a short-period range of 6–20 Hz were obtained using three different methods: the 6C Rotaphone sensor system developed by the authors, the commercial R-1 rotational sensor by Eentec, and a small-aperture array of twelve standard velocigraphs in a rectangular arrangement. Those three methods are compared and discussed in detail. A medium-size quarry blast was used as a seismic source. At a distance of approximately 240 m, the rotation rates reached an amplitude of the order of magnitude of 10${}^{-4}$–10${}^{-5}$ rad/s. The array derived rotation rates displayed serious limitations, as clearly documented. The R-1 instruments have shown certain technical problems that partly limit their applicability. The measured rotation rates were compared to the relevant acceleration components according to rotation-to-translation relations. Out of all the three methods, the records best matching the acceleration components were made by Rotaphone. The experiment also revealed that rotation rates in the given short-period range noticeably changed over a distance as short as 2 m.

## 1. Introduction

**Figure 1:**
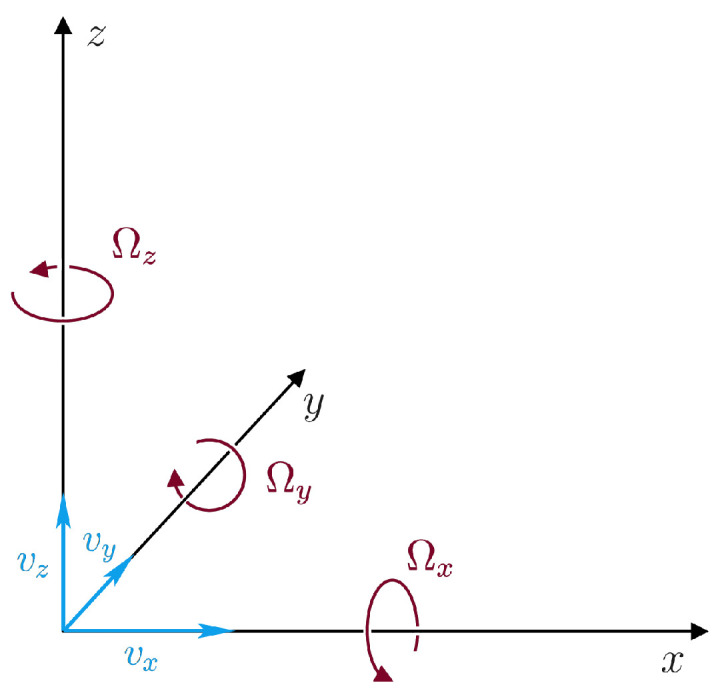
Sign conventions for translational and rotational components used in this study. The *x*-axis points to the North.

Observational seismology mainly relies on measuring translational ground motions by traditional seismometers. Here, by translational motions, we mean the three Cartesian components of ground velocity v (Figure 1), as measured by conventional inertial seismographs that consist of a mass attached to a fixed frame. Another kind of seismic instruments, known for decades, are strainmeters [1], which are used to measure the deformation of the Earth by detecting changes in the distance between two points. However, there is a third type of seismic motion, seismic rotations [2], which should be measured together with translations and strains in order to obtain a complete description of the seismic wavefield in a close vicinity of an observation point. A growing interest in detecting and interpreting seismic rotational motions gave rise to new seismological disciplines, rotational seismology and rotational seismometry, and it has been demonstrated by the scientific journals publishing several special issues exclusively dedicated to these new disciplines (examples are: Bulletin of the Seismological Society of America [3], Journal of Seismology [4], and the present special issue of the Sensors journal).

Rotational seismology deals with rotational ground motions from earthquakes or other seismic sources. The rotational ground motion can be decomposed into three rotational components. In this paper, we focus on measuring rotation rate Ω, related to the curl of ground velocity v. At the Earth’s surface, thanks to the free-surface boundary conditions, the rotation rate components simplify to

(1) $$\begin{array}{ccc} \Omega_{x} & = & \frac{\partial v_{z}}{\partial y}\\ \Omega_{y} & = & -\frac{\partial v_{z}}{\partial x}\\ \Omega_{z} & = & \frac{1}{2}\left(\frac{\partial v_{x}}{\partial y}-\frac{\partial v_{y}}{\partial x}\right), \end{array}$$

where x,y,z refer to a right-handed Cartesian coordinate system (Figure 1). The $\Omega_{z}$ component (rotation rate around the *z*-axis) is sometimes called the torsion rate, while the other two components (rotation rates around the horizontal axes) are traditionally called tilt rates.

The development of rotational seismology has been made possible by reliable measurements of seismic rotations. For that reason, the focus of rotational seismology now lies on the development of rotational instruments and corresponding measuring methods.

Rotational seismometry has been boosted in the 2000s with the onset of the ring laser technology that is based on the Sagnac effect [5]. Nowadays, ring-laser gyroscopes are able to measure absolute rotational motions, induced by teleseisms, with rates reaching the order of magnitude as little as $10^{-12}$ rad/s [6]. Despite of such diminutive values, seismic rotation represents a new observable that is worth studying, measuring, analyzing, and interpreting in all scales of epicentral distances. In combination with translational data that are produced by a single-point collocated measurement, seismic rotational components have shown to be capable of providing information on the subsurface structure, in terms of apparent phase velocity of seismic waves [7], as well as propagation direction, i.e., the true back azimuth from which the waves came [8]. Kurrle et al. [9] applied subsequent narrow band-pass filters with a growing central frequency to ring laser data and examined the possibility of estimating Love wave dispersion curves from single-station measurements. Ring-laser rotational measurement helped to identify Love-wave energy in the secondary microseism [10]. Rotational components may also be useful for better understanding of the physics of earthquake source through more constrained inversion of seismic source parameters [11].

With an increasing number of papers dealing with the possibility to retrieve phase velocity of seismic waves by employing collocated rotation and translation records, a question arose as to the depth resolution of the corresponding methods, i.e., down to what depth the velocity can be recovered in this way. By analyzing sensitivity kernels at long periods in certain global one-dimensional (1-D) Earth models, it has been found that the resolvable subsurface volume is highly localized below the receiver position and the sensitivity of these techniques is restricted to shallow depths not exceeding about one wavelength of the studied wavefield [12,13]. Brokešová and Málek [14] came to the same conclusion when studying short-period synthetic data at a small epicentral distance from a shallow point source in a 1-D model that was overlaid by a thin low-velocity subsurface layer. Such results indicate that, in all scales, apparent seismic phase velocities retrievable from collocated rotational and translational measurements are sensitive to the near-receiver structure rather than the structure along the whole wave path. What one might consider as a limitation in fact opens up wide-spread applications of rotational seismology (and seismometry) in seismic prospecting and engineering seismology.

Ring lasers are highly sensitive, but they require a very costly installation, operation, and maintenance, and they cannot be used in routine field measurements. Therefore, it has become necessary to develop small portable flexible instruments that are easily deployable in the field in a fast response to the current seismic situation or in active experiments used in seismic exploration and engineering. To address that need, various rotational sensors have been developed on both a commercial and non-commercial basis, especially during the last decade. Some of them, such as fiber-optic gyroscopes, utilize the Sagnac effect similarly as the ring lasers. Examples are BlueSeis3A [15] and AFORS [16]. Others, like R-1 and R-2, by Eentec [17], are based on electrochemical technology. Applied Technology Associates (ATA) has developed a proto-seismic magnetohydrodynamic rotation-rate sensor that proved to be applicable in recording seismic rotations from micro-earthquakes at local distances [18]. We have applied a completely different principle and developed a mechanical sensor system, called Rotaphone. It is based on measuring differential motions between paired sensors mounted on a rigid frame anchored to the ground. The elemental sensors themselves measure seismic translational motions. The differential motions, which represent spatial ground motion derivatives, are used to derive seismic rotational components. In this way, the instrument is capable of collocated measurement of both seismic translations and rotations with the same device, i.e., influenced by the same instrument characteristics.

Emerging portable rotational sensors, which were developed by different scientific groups or companies, have to not only undergo thorough tests in laboratories and in the field, but they should also be subjected to experiments aimed at their mutual comparison. As an overwhelming majority of seismic rotational data are records of motions very weak in amplitude, such experiments should be focused on that rather than on strong motion records. To verify Rotaphone data for weak events, we designed a comparative in-field experiment (inspired by the paper by Kendall et al. [19]). The experiment consisted in comparing Rotaphone records with those from the Eentec R-1 rotational sensor and the array-derived-rotation (ADR) data. A medium-size blast at the Klecany quarry near Prague (Czech Republic) was used to generate seismic waves. The results that were obtained from this blast are provided below. As far as the authors know, the experiment presented here was the first in which more than one type of portable seismic rotational sensors were involved. Only recently, at the end of 2019, another comparative sensor test experiment was organized at the Geophysical Observatory of the LMU in Fürstenfeldbruck (Germany). That experiment involved more than 40 different sensors and technologies that were capable of measuring weak seismic rotations and the results are expected to be published in the near future.

Figure 1 shows the coordinate system and axes orientation used for the individual components in this study. In contrast to the R-1 sensor by Eentec (Section 2.1), the rotation rates are positive counter-clockwise in accordance with the right-handed ‘rule of thumb’. Note that this orientation agrees with the suggestion made by Evans [20].

### Quarry Blast Experiment Setup

The experiment took place near Prague, at the Klecany quarry, on a sunny day with a mild temperature of ∼20 ${}^{\circ }$C. The prevailing rock in the quarry is greywacke. The test site was a horizontal plot on one of the floors created after mining out the rocks. It was situated approximately 240 m away from the blast site and the back azimuth was about 320 degrees from the North. The explosive weight was 1400 kg.

**Figure 2:**
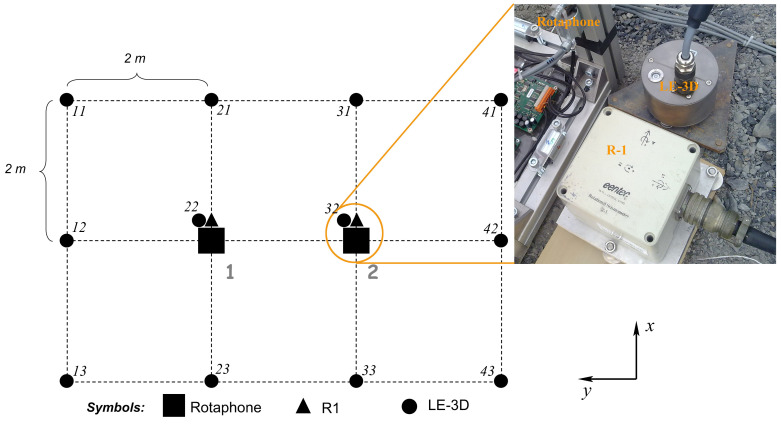
The comparative experiment — basic instrument lay-out. Two-digit numbers code LE-3DLite velocigraphs. Grey numbers mark two central grid points. Inset: one of the Eentec R-1 rotational sensors in the experimental setting together with the Lennartz LE-3DLite translational sensor and the Rotaphone sensor system.

Figure 2 shows the basic instrument lay-out in the experiment. We used twelve short-period Lennartz LE-3DLite velocigraphs (loaned from the Institute of Geophysics, CAS) arranged in a rectangular-grid scheme (3 × 4) with the separation distance of 2 m. The longer side of the array was oriented in the West–East direction. In the middle of the grid, there were two central points, marked as 1 and 2 in the figure, equipped with single point seismometers capable of measuring seismic rotations: the six-component Rotaphones developed by the authors and the commercial R-1 sensors (kindly provided by Dr. Chin-Jen Lin from the Institute of Earth Sciences, Academia Sinica, Taipei, Taiwan). The experiment was designed to compare rotational records at the central points 1 and 2. For that reason, the instruments at the central grid points were posed as close to each other as possible (Figure 2, inset). The inset figure also shows the character of the bedrock.

## 2. Methods That Were Used to Determine Seismic Rotation

### 2.1. R-1 (Eentec) Measurement at a Point

One of the methods used in the comparative experiment is a direct measurement with the commercial Eentec R-1 rotational sensor. It is a triaxial rotational velocity sensor that is based on electrochemical technology. The sensor uses the principle that ground motion induces motion of electrolytic fluid inside a torus. The fluid motion is transduced to a voltage signal that is sensed. The output voltage is proportional to the rotation rate around the axis of the torus. The tri-axial device contains three elemental electrochemical transducers arranged in three perpendicular directions. The dimensions of the device are 12 cm × 12 cm × 9 cm and it weighs 0.9 kg. Figure 2 (inset) shows the R-1 sensor in an experimental setting together with the Lennartz LE-3DLite 1 Hz seismometer (Section 2.3.1) and a part of our Rotaphone sensor system (Section 2.2). It is apparent from the figure that the R-1 sensor uses an unusual left-handed coordinate system for the direction of positive rotation. The sensor is capable of recording small earthquakes at distances up to several tens of kilometers. Table 1 (top) lists the specification provided by the manufacturer.

**Figure 3:**
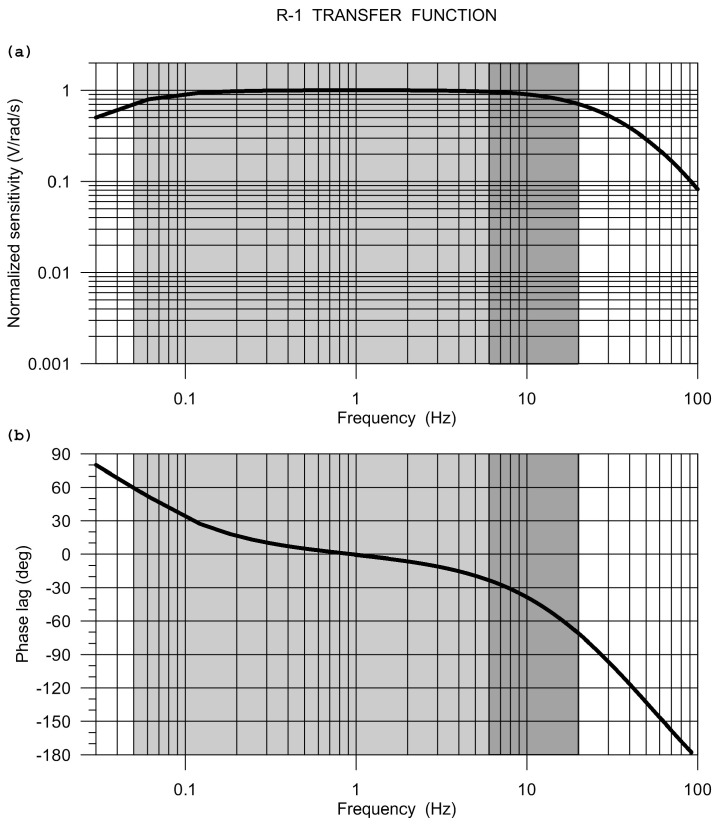
The Eentec R-1 frequency response: modulus (**a**) and phase (**b**). The response is calculated from poles and zeros specified by the manufacturer. Grey intervals indicate the specified frequency range, the dark grey rectangles mark the range actually considered in the comparative experiment.

The Eentec R-1 frequency response, as calculated from the manufacturer-provided pole-zero model, is shown in Figure 3.

The sensor has been rigorously and independently tested by research teams in the United States, Taiwan, and Germany. Extensive tests that were carried out by Lin et al. [21] and Nigbor et al. [17] led to the conclusion that the R-1 sensor meets the specifications given by the manufacturer only generally. The measured sensitivity values deviated from the nominal factory specification by as much as 30% [21]. Nigbor et al. [17] confirmed the hard-clip level (maximum voltage output regardless of input), but they have found that there is a soft-clip level that is represented by the amplitude at which the output begins to be distorted or nonlinear. The tests revealed an average soft-clip level of about 75% of the full scale at 1 Hz. At low frequencies, the measured self-noise was at least by one order of magnitude higher than that specified by Eentec. The frequency response shape also deviated a little from the declared response, both in magnitude and phase. The judgment derived from the tests is that the R-1 sensor is able to provide reasonable rotational data, but sensor-specific calibration should be considered to increase confidence. Moreover, it has been found that the R-1 rotational sensor is very sensitive to temperature [22].

As observed by Nigbor et al. [17] and confirmed by Lin [23], the R-1 sensors require several minutes to stabilize from a hard clip, such as when the sensor is moved or tilted. This behavior may also limit the applicability of the R-1 sensors in near-source regions where strong ground motion could be expected. Problems of this kind might complicate the comparative experiment, as the considered blast was launched five minutes after a preceding strong shot.

We performed some preliminary laboratory experiments with the R-1 sensors and found that the given sensors could have problems at low frequencies (mutual inconsistencies when the sensors were placed next to each other, 11 cm apart). Therefore, we decided to filter out the low-frequency part from the records. As the upper frequency limit for the R-1 sensors is declared as 20 Hz, we compared the records from the three methods (Rotaphone, R-1, and ADR) only in the frequency window 6–20 Hz.

### 2.2. Rotaphone Measurement at a Point

Rotaphone is a mechanical sensor system (or shortly sensor) that is capable of collocated measurement of both ground velocity and rotation rate with one device [24,25]. It exists in various designs, all of them being based on the same principle. Highly sensitive geophones, which are used to record short-period translational components, are mounted in parallel pairs to a rigid (metal) ground-based frame. Such an arrangement makes it possible to measure, in addition to translations, also differential motion between the paired geophones. Those differential records are used to obtain rotational components via equations

(2) $$\Omega_{x}=\frac{\partial v_{z}}{\partial y}=-\frac{\partial v_{y}}{\partial z},\;\Omega_{y}=\frac{\partial v_{x}}{\partial z}=-\frac{\partial v_{z}}{\partial x},\;\mathrm{and}\;\Omega_{z}=\frac{\partial v_{y}}{\partial x}=-\frac{\partial v_{x}}{\partial y}.$$

The simplification in the above equations is not due to the presence of the Earth’s surface, but, primarily, due to the rigidity of the frame, which, being set to motion by the ground motion, moves as a rigid body and the geophones measure this rigid-body motion. Thanks to the rigidity, strain components vanish and the rotational rate components are simplified due to corresponding constraints that were applied to the relevant spatial gradients.

Thus, to obtain rotational components we need to subtract the records from the geophones of a given pair (and divide the difference by the separation distance), while to obtain translational components we sum the records of co-axial geophones and take the average. The separation distance in a pair is typically a few tens of cm, i.e. the distance is much smaller (at least by two orders of magnitude) than the wavelength typically considered, but still large enough to allow differential sensing. Note that the rotational components obtained by differencing the records from the paired geophones are translation insensitive. The reason is that the translational components are subtracted from the rotational records, because, thanks to the rigid body motion, the paired geophones are subject to the same translation. The horizontal translational data from Rotaphone are corrected for contamination by tilts [26] as part of the data processing. Thus, translational data are rotation-free.

**Figure 4:**
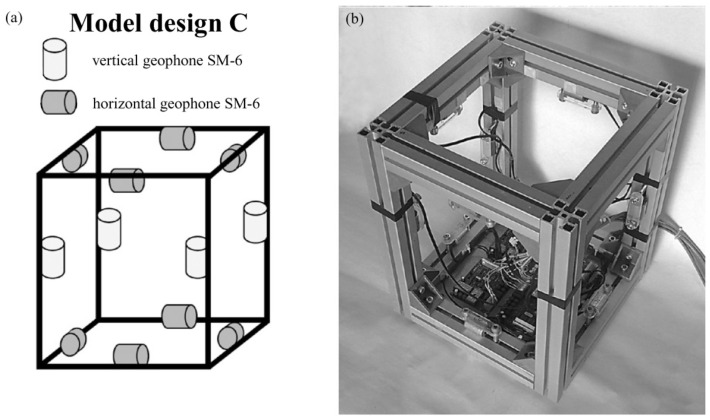
Rotaphone-C: scheme of geophone pairs (**a**) and photograph (**b**).

The Rotaphone design that is involved in the comparative experiment is called Rotaphone-C and it is shown in Figure 4. It consists of eight horizontal and four vertical geophones SM-6 (Ion) mounted onto a cubic-shaped metal frame 35 cm × 35 cm × 43 cm. The separation distance between the paired geophones is 30 cm. The geophones are connected to a 28-bit A/D convertor. Rotaphone-C has been subjected to specialized testing at the USGS Albuquerque Seismological Laboratory, New Mexico, USA. Some of the results of those tests are reported in the paper by Brokešová et al. [27]. Besides several-months lasting measuring campaigns in the West Bohemia/Vogtland earthquake swarm region (Czech Republic) and in the active opening rift region of the Gulf of Corinth (Greece), it has been successfully applied since 2013 in a continuous monitoring in the vicinity of the Katla volcano, South Iceland [28,29]. Table 1 (middle) specifies the parameters of the instrument. The upper frequency limit is given by the frame’s natural frequency (first resonance mode frequency), because only up to this frequency the frame moves as a rigid body, which is a requisite feature of our approach. The lower limit is determined by the behavior of the given geophones at low frequencies: it is the frequency below which the geophone output starts to be distorted compared to the geophone input. The resolution values represent the noise-free smallest detectable ground motions. In real measurements, noise is always present and the smallest detectable motions are considerably higher. The smallest rotation rates really recorded by Rotaphone-C are of the order of magnitude of 10${}^{-8}$ rad/s in a low-noise environment.

**Figure 5:**
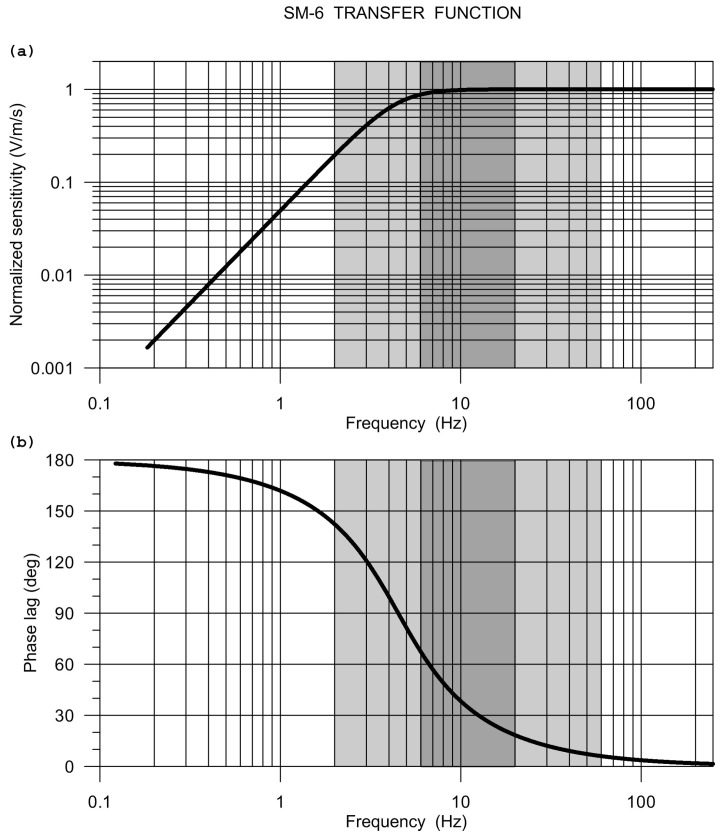
The SM-6 frequency response: modulus (**a**) and phase (**b**). The response is calculated from poles and zeros specified by the manufacturer. Grey intervals indicate Rotaphone frequency range, the dark grey rectangles mark the range actually considered in the comparative experiment.

Figure 5 shows the SM-6 transfer function that was calculated from poles and zeros provided by the manufacturer. As rotational components come from translation differences, the same normalized transfer function is valid for both rotational and translational records. This is a great advantage when studying rotation-to-translation relationships.

The main problem in measuring rotational components by differencing records from proximal parallel geophones is that the geophones are not entirely identical in their characteristics and, therefore, they are not able to produce identical output for the same input. This results in a relative error, which, although possibly small in itself, can substantially affect the small differential motion. The only way to solve the problem is to calibrate the individual geophones as precisely as possible. It is usually not sufficient to calibrate the geophones once in a lab, because their characteristics depend on the current physical conditions (temperature, pressure, magnetic field variations, etc.) and aging of the geophones. Hence, it is necessary to calibrate them on an ongoing basis, simultaneously with each measurement (in situ). Rotaphones enable such calibration, as they have more than one geophone pair for each rotation rate component, e.g., the Rotaphone-C model design provides four pairs for each of them. Ideally, thanks to the rigidity of the frame, the same rotational rate component should be obtained from all of the relevant pairs. This multivaluedness yields constraints that enable a precise calibration during data processing. The basic principle underlying our calibration technique was briefly described [24,25]. The ‘in-situ’ calibration of the individual geophones is an essential part of each Rotaphone measurement. It is necessary to achieve the required accuracy and sensitivity.

Summarized, Rotaphones are short-period seismic instruments that provide both translational and rotational records. Rotational components are free of translations and the translational components are free of rotations. Detectable amplitudes go down to the order of magnitude of $10^{-7}$ rad/s (for a very low noise level, even $10^{-8}$ rad/s) and $10^{-8}$ m/s. The maximum measurable amplitude, as given by the geophone’s hard-clip level, is of the order of magnitude of $10^{-1}$ in detecting both the translational (m/s) and rotational (rad/s) motions. Thus, these instruments can be used to measure amplitudes in a relatively wide range of both very weak and strong (except for extremely strong) ground motions. An important feature of the Rotaphone measurement is the ‘in-situ’ calibration performed simultaneously with each measurement (as an integral part of data processing). This approach ensures that aging of the sensors or changing physical conditions do not influence the instrument characteristics.

The rigid connection of the sensors, the extremely small separation distance between them, and their in-situ calibration distinguish Rotaphone the most substantially from the ADR method (Section 2.3.2), which is also based on detecting differential motions.

### 2.3. Array Measurement

#### 2.3.1. Small-Apperture Array of LE-3DLite Sensors

**Figure 6:**
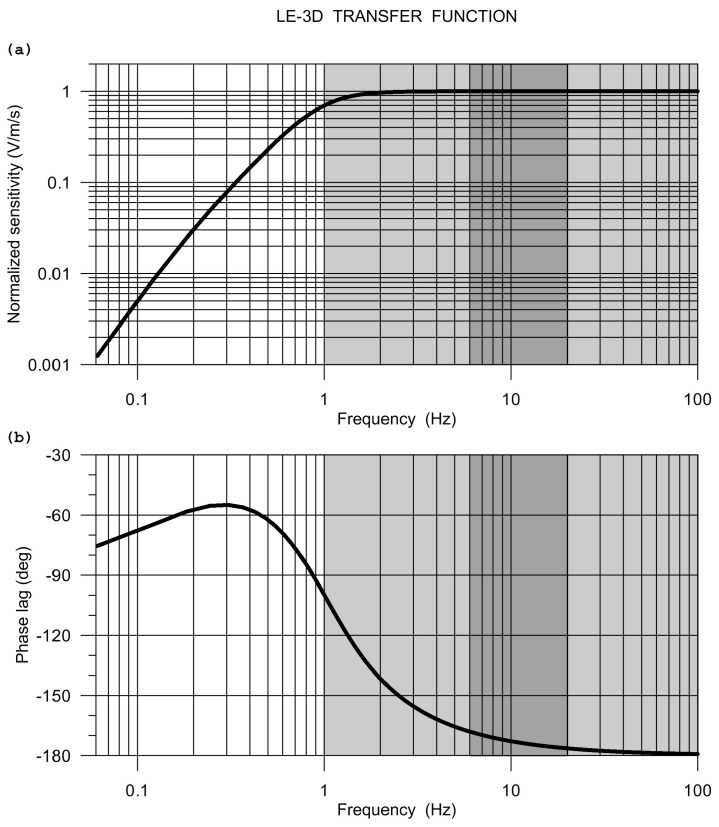
The Lennartz LE-3DLite frequency response: modulus (**a**) and phase (**b**). The response is calculated from poles and zeros specified by the manufacturer. Grey intervals indicate the specified frequency range, the dark grey rectangles mark the range actually considered in the comparative experiment.

**Figure 7:**
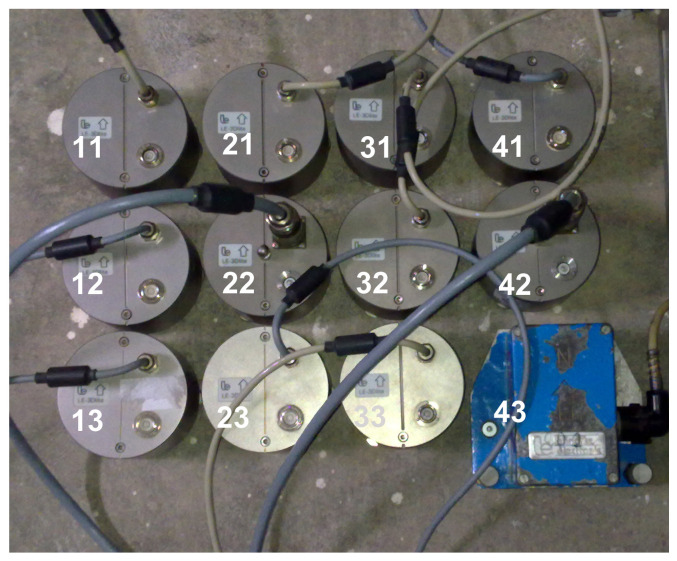
The Lennartz LE-3DLite huddle test lay-out. The centers of the sensors are arranged in a rectangle of 22.5 cm × 31.5 cm.

**Figure 8:**
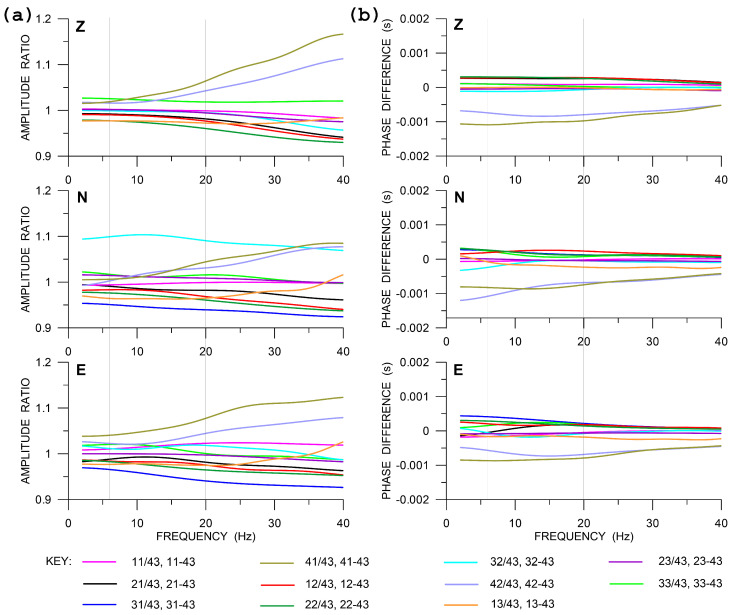
Amplitude ratios (**a**) and phase differences (**b**) of the individual LE-3DLite sensors with respect to the reference one for vertical (Z), North (N) and East (E) components. The thin vertical grey lines mark the frequency range actually considered in the comparative experiment. Two-digit sensor identifiers shown in Figure 7.

In the comparative experiment, we deployed a small-aperture rectangular array (Figure 2) of 12 standard short-period LE-3DLite velocigraphs by Lennartz Ltd. These are compact three-component seismometers in a cylindrical housing with a diameter of 97 mm and height of 68 mm. The small dimensions are convenient when installing more instruments at one site, like in the case of the two central points in Figure 2 or during a huddle test (see below). The LE-3DLite seismometer is based on three orthogonal 4.5 Hz geophones with the eigenfrequency decreased to 1 Hz. The parameters of the instruments, which are relevant for the experiment, are specified in Table 1 (bottom). The specified transfer function, as calculated from poles and zeros provided by the manufacturer, is shown in Figure 6. In contrast to Rotaphones, the velocity transfer function that is shown in Figure 6 is not easily related to the rotation rate transfer function, as here is no rigid connection between the individual sensors of the array and the in-situ calibration technique described above cannot be applied. Therefore, in order to reveal any possible differences in the actual responses of the individual sensors, a huddle test was performed, in which the sensors were installed in one place as close to each other as possible (Figure 7) in a relatively high noise environment to record seismic signal for several hours overnight. The spectra that were smoothed by a moving window in time were used to find corrections for at least the boldest differences among the actual frequency responses of the individual seismometers in the array. The huddle test indicated that those differences are significant (Figure 8); in the frequency range of interest (6–20 Hz), we found amplitude differences reaching almost 10% and phase differences up to 1 ms. During the comparative experiment, we had no time to conduct a systematic laboratory calibration measurement that would yield a detailed actual transfer function of each LE-3DLite sensor involved.

#### 2.3.2. Array Derived Rotation Method

In the ADR method, rotational components are derived from differential motions that were obtained by subtracting the records from close stations in a seismic array. When the receivers in the array are laid out in right angles, local spatial gradients along the two perpendicular directions can be easily approximated by taking finite differences between records from neighboring receivers in the given directions [30]. The only condition for obtaining sufficiently accurate approximations of the spatial gradients (rotational components) in this way is a sufficiently small separation distance between the receivers with respect to the wavelength of the studied wavefield. Various authors may set the criteria controlling the separation distance in a slightly different way (according to the required accuracy in specific applications). In general, such criteria are similar to those that were adopted to control grid spacing in the finite-difference method for wavefield modeling. For example, a widely accepted criterion for second-order finite-difference schemes requires the grid spacing not to exceed ∼1/10 of the shortest wavelength in the wavefield. Of course, as the arrays are usually spread on the Earth’s surface, the wavelength here is understood as the apparent wavelength along the surface. In the presence of noise (i.e., in real measurements), such a criterion should be thoroughly examined, as explained below.

**Figure 9:**
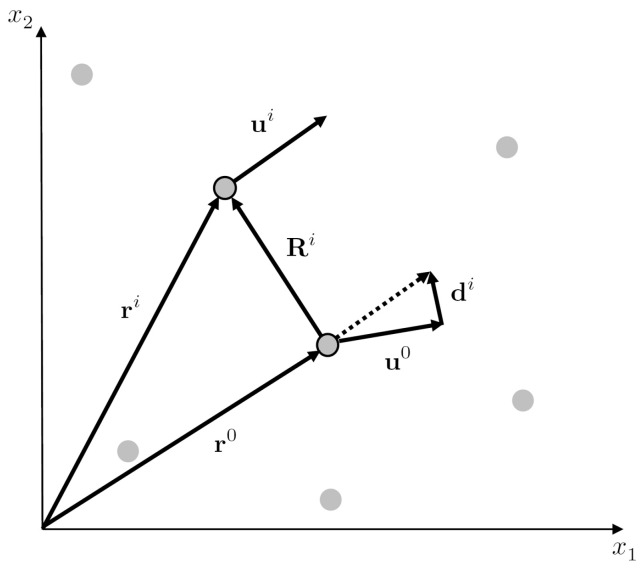
A scheme of an array in a general layout with definitions of the vectors used in the array-derived-rotation (ADR) method.

This approach can be generalized for general array layouts (Figure 9). The problem is linear under the assumption of uniform spatial gradients of the first order across the array. Assuming uniform first-order gradients, we, in fact, assume that higher-order gradients vanish. Subsequently, we can, in principle, determine the first-order gradients from the Taylor’s expansion up to the first order [31]

(3) $$v_{i}\left(x+\delta x\right)\approx v_{i}\left(x\right)+\frac{\partial v_{i}\left(x\right)}{\partial x_{j}}\delta x_{j}.$$

Let us explain the approach while employing the same notation as in that paper, except for ground velocity v considered instead of displacement u. Assume a reference receiver in the array, characterized by the predisturbance position vector $r^{0}$. Let $r^{i}$, i=1,…N be the predisturbance position vectors of the remaining *N* receivers in the array. Figure 9 shows the reference receiver and one such receiver at a position $r^{i}$ selected from among the others. Let us denote ground velocity at the reference receiver by $v^{0}$, i.e., $v^{0}=v\left(r^{0}\right)$. Similarly, $v^{i}=v\left(r^{i}\right)$ is velocity at the *i*-th receiver. In the figure, $R^{i}$ denotes the position vector between the reference and the *i*-th receiver. This vector is an analog of δx in Equation (3). In accordance with Equation (3), the differential motion $d^{i}=v^{i}-v^{0}$ between the reference and *i*-th receiver (which is close to the reference one) satisfies the equation

(4) $$d^{i}=GR^{i},$$

where G is a 3 × 3 ground velocity gradient matrix with the elements $G_{ij}=v_{i,j}$, which are to be determined, as they constitute the curl of v, the quantity of our primary concern. Note that no upper index is necessary for these elements thanks to the uniform spatial gradients assumption.

The problem of spatial gradient determination is simplified for an array on the Earth’s surface (the most common case in seismological practice). Assume that the surface coincides with the plane x,y in a right-handed Cartesian coordinate system x,y,z with *z* being the vertical. All of the vectors $r^{0}$, $r^{i}$, and $R^{i}$ (i=1,…N) lie in this plane, i.e., their vertical components vanish. Moreover, the free-surface conditions (vanishing stress along *z*) yield constraints for certain elements of matrix G so that only six of them are independent. Written out into components, Equation (4) then becomes

(5) $$\left(\begin{array}{c} d_{x}^{i}\\ d_{y}^{i}\\ d_{z}^{i} \end{array}\right)=\left(\begin{array}{ccc} v_{x,x} & v_{x,y} & -v_{z,x}\\ v_{y,x} & v_{y,y} & -v_{y,x}\\ u_{z,x} & v_{z,y} & -\frac{\lambda }{\lambda +2\mu }\left(v_{x,x}+v_{y,y}\right) \end{array}\right)\left(\begin{array}{c} R_{x}^{i}\\ R_{y}^{i}\\ 0 \end{array}\right).$$

As $R_{z}^{i}=0$, it is not possible to determine the third column of G without knowing the structure in terms of the Lamé coefficients λ and μ. Fortunately, to determine the curl of v, we need only $v_{z,y},v_{z,x},v_{x,y}$ and $v_{y,x}$, not the whole matrix G. Equation (5) can be rewritten when considering the 2 × 3 left submatrix of G

(6) $$\left(\begin{array}{c} d_{x}^{i}\\ d_{y}^{i}\\ d_{z}^{i} \end{array}\right)=\left(\begin{array}{c} v_{x}^{i}-v_{x}^{0}\\ v_{y}^{i}-v_{y}^{0}\\ v_{z}^{i}-v_{z}^{0} \end{array}\right)=\left(\begin{array}{cc} v_{x,x} & v_{x,y}\\ v_{y,x} & v_{y,y}\\ v_{z,x} & v_{z,y} \end{array}\right)\left(\begin{array}{c} R_{x}^{i}\\ R_{y}^{i} \end{array}\right).$$

The left-hand side of the equation is obtained by subtracting the corresponding velocigrams. For the *i*-th receiver, the equation represents a system of three linear equations for six unknowns, the elements of the matrix on the right-hand side, of which $v_{x,x}$ and $v_{y,y}$ are redundant for obtaining rotational components in Equation (1). Therefore, we have to write down the above equation for at least two receivers with position vectors $r^{k}$ and $r^{l}$, which means that minimum three stations that are equipped with three-component translational sensors should be used (together with the reference receiver $r^{0}$) in the ADR method in order to approximate the complete rotation rate vector. In practice, however, it is commendable to consider as many stations as possible, as that compensates for inconsistencies in the array measurement (variations in instrument characteristics, in soil conditions underneath the individual receivers, etc.). For this reason, Equation (6) should be solved for many stations, even for arrays with a rectangular layout because calculating spatial gradients (finite differences) from the closest neighbors of the target point may be very inaccurate.

As said above, Equation (6) is applicable only if the spatial-gradient waveforms do not vary across the array. This is the principal assumption of the ADR method. The requirement of the uniform gradient distribution naturally leads to limits for the array size, as discussed in detail by Donner et al. [32]. A non-uniformity of the measured spatial gradients may be due to both physical and instrument-related reasons. The above-mentioned inconsistencies in the characteristics of the instruments that are involved in the array requires a low-frequency (long-period) limit, as explained in Langston et al. [33] or Poppeliers and Evans [34]. Such a limit is not an issue in our study, as we work in a short-period range. As regards a high-frequency limit, Spudich et al. [31] did not set any quantitative criterion, saying just that “the array …(should span)… only a small fraction of a wavelength in the period range of interest”. Spudich and Fletcher [35] suggested that the horizontal extent of the array, parallel to the wave propagation direction, should not exceed one-quarter of the apparent wavelength measured along the Earth’s surface. Note that even at the highest frequency of 20 Hz considered in this study, the Spudich–Fletcher’s criterion for the array size in the ADR method is amply satisfied for a conservative estimate of typical S-wave velocity at a basalt-quarry site (1–2 km/s). However, for the wavefield from proximal sources, the assumption of uniform spatial gradients is often difficult to satisfy, even if the wavelength complies with the criterion by Spudich and Fletcher. Therefore, at a small source-array distance, the fulfillment of the gradient uniformity assumption should be of a higher priority than any formal criteria set on the wavelength, which can, moreover, only be estimated in most cases. Based on our experience with quarry-blast experiments, the uniformity of the gradients should be checked before the application of the ADR method whenever possible.

Looking beyond the above mentioned problems with possibly inconsistent measurement by the individual sensors in the array and non-uniform gradients across the array, there are at least two other crucial issues to be discussed regarding the ADR method. The first concerns the separation distance between the individual receivers. On one hand, the smaller it is, the better approximation of spatial derivatives can be achieved by finite differencing. On the other hand, as mentioned by Cochard et al. [36], the smaller the distance, the more similar the records are and, consequently, the smaller differential motion that we obtain from the given receivers. In the presence of noise, both external and instrument-related, the signal-to-noise ratio for the differential motion from stations too close to each other may become so indisposed towards the spatial gradient determination that it may degrade the ADR technique altogether. Suryanto et al. [37] studied the effect of noise on both synthetics and on real data, and suggested that this problem can be partly overcome by including more stations in the ADR approach, so that the uncorrelated random noise may cancel out.

Another problem arises from the fact that the measured horizontal components in Equation (6) may not be ‘pure’ translations because of contamination with tilts [26]. While, at higher frequencies, this contamination is negligible, at low frequencies, which the ADR approach is limited to, it may be significant. It may lead to incorrect results when these contaminated horizontal translations are used to derive the rotational components.

To summarize, although the ADR technique has a very simple concept and it does not require complicated computation, it suffers from serious problems accompanying it. It should be used with greatest care, even after verifying the basic assumptions, optimizing the frequency range and/or separation distance, checking the noise level, taking into account the uncertainty in seismometer calibration, site effects, possible contamination with tilts, etc.

## 3. Results

### 3.1. Velocity Records and Velocity Spatial Gradients

**Figure 10:**
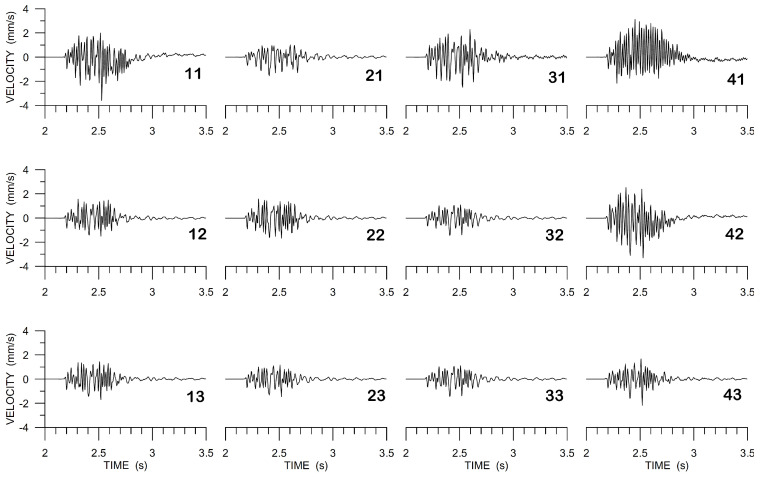
Array raw data (LE-3DLite), E-component. The arrangement of the seismograms corresponds to the array arrangement in Figure 2. Two-digit numbers are used to code the individual stations.

**Figure 11:**
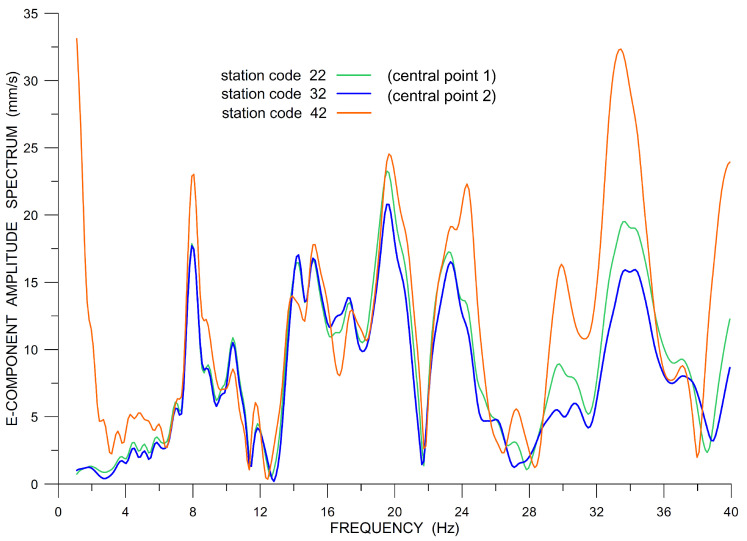
Array raw data (LE-3DLite), examples of amplitude spectra at three selected stations, E-component.

The following three figures illustrate some of the problems with ADR that we encountered, with a focus on the E-component chosen as an example. The other two components suffered analogous problems. Raw data that were recorded within the array (E-component, back azimuth 320${}^{\circ }$ from the North) are shown in Figure 10. It can be clearly seen that some of the data show instrument disturbances that can be interpreted as the instrument response to a step in input acceleration or velocity [38]. Although they are seemingly low-frequency disturbances, they contaminate the records over all frequencies, as it is demonstrated in Figure 11 when comparing unfiltered amplitude spectra at the two central grid points (stations 22 and 32) with the spectrum from the rightmost station in the middle row (42). While the two spectra at the central points (green and blue curves) are very close to each other up to ∼ 16 Hz and deviate only slightly towards higher frequencies (within the considered frequency window), the spectrum of the third station (the one suffering from the instrument disturbance) deviates from the other two much more in the whole interval of 6–20 Hz. Therefore, stations with such problems were excluded from the processing.

**Figure 12:**
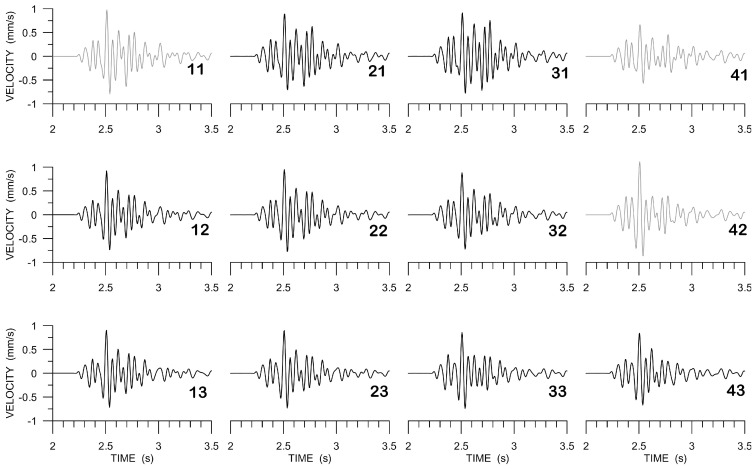
Array data (LE-3DLite), bandwidth filtered (6–20 Hz), and instrument-corrected, E-component. The arrangement of the seismograms corresponds to the array arrangement in Figure 2. Two-digit numbers are used to code the individual stations. Seismograms excluded from further processing are shown in grey.

Figure 12 shows the E-component array data that were filtered from 6 to 20 Hz, instrument-corrected (also considering small variations between the individual instruments measured in the laboratory during the huddle test, see above), and corrected for possible inconsistencies in station azimuths (small differences of the order of magnitude of degrees were corrected by correlating the very beginning of the horizontal-component data from station to station at low frequencies). The records used in further processing are shown in black, while the excluded data in grey.

**Figure 13:**
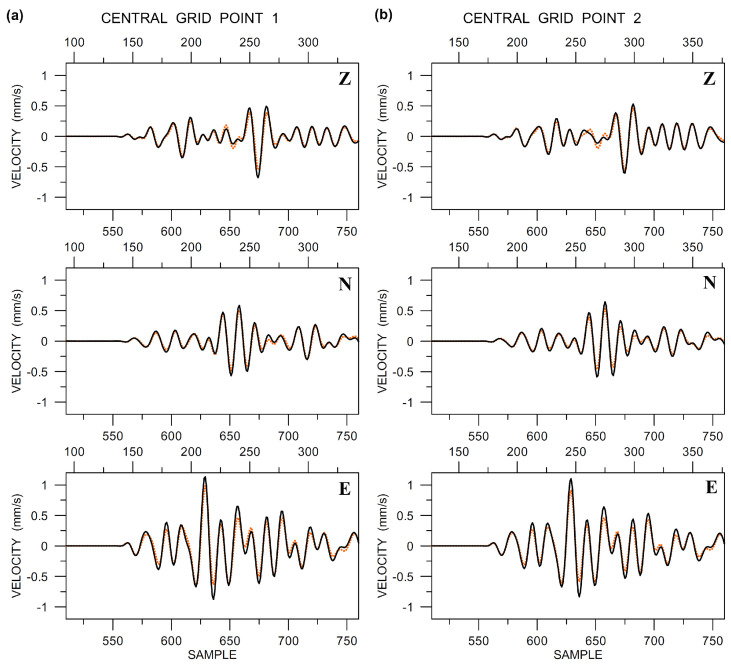
Comparison of the LE-3DLite (dotted orange) and Rotaphone (solid black) translational components (vertical Z, North N, and East E) at the central grid points 1 (**a**) and 2 (**b**). Data are band-pass filtered between 6 and 20 Hz and instrument-corrected. The sampling frequency was 250 Hz.

It is interesting to see the translational components that were measured by the LE-3DLite sensors at the two central points, compared with those measured by Rotaphones in the same place (Figure 13). Both types of data are band-pass filtered by the same causal Butterworth filter between 6 and 20 Hz and instrument-corrected using the instrument response provided by the corresponding manufacturer. The characteristics of that response may slightly differ from the actual responses of the particular instruments that are involved in the comparison, which may be the cause of slight differences in amplitudes between the LE-3DLite and Rotaphone records. Note that the Rotaphone translational data that are shown in the figure are, in addition to standard instrument correction, also corrected using the ‘in-situ’ calibration technique that suppresses inconsistencies in the actual transfer functions of the sensors in the system and equalizes all of them with the transfer function of the reference sensor. However, the reference-sensor transfer function may slightly deviate from the actual response, which may affect the Rotaphone data in a similar way as the LE-3DLite data. Also note slight differences in the translational records between the two central points, which can be seen in Figure 13, most prominent on the *z*-component.

**Figure 14:**
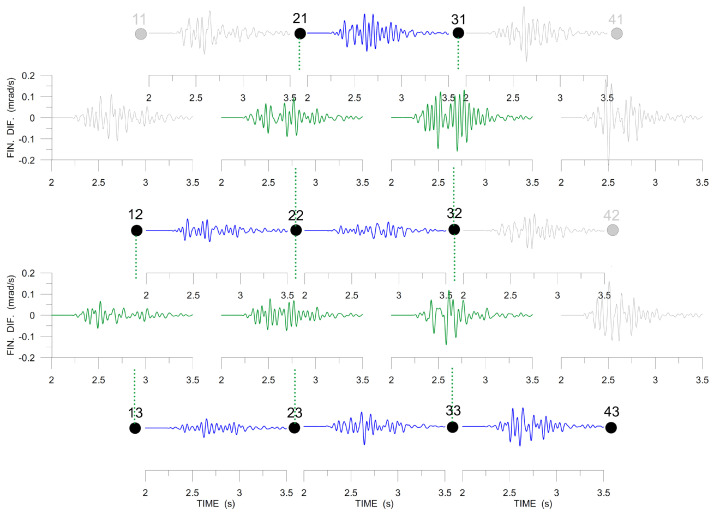
Band-pass filtered (6–20 Hz) finite differences, $\Delta v_{x}/\Delta y$ (blue), $\Delta v_{y}/\Delta x$ (green), *x*-axis pointing to the North, *y*-axis pointing to the West. The large dots mark the stations in the array (see Figure 2). The differential seismograms are drawn between the dots corresponding to the stations used to derive them. The data excluded from further processing are shown in grey.

**Figure 15:**
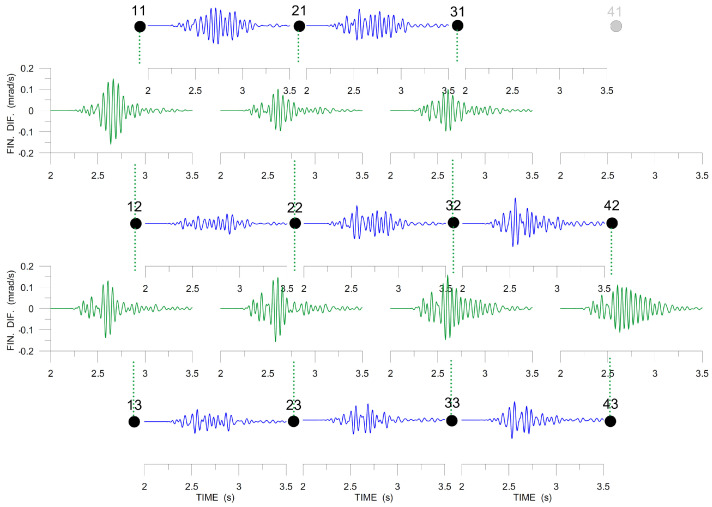
Band-pass filtered (6–20 Hz) finite differences, $\Delta v_{z}/\Delta x$ (green), $\Delta v_{z}/\Delta y$ (blue), *x*-axis pointing to the North, *y*-axis pointing to the West. The large dots mark the stations in the array (see Figure 2). The rightmost station at the top indicated by grey for missing *z*-component data. The differential seismograms are drawn between the dots that correspond to the stations used to derive them.

The next two figures, Figure 14 and Figure 15, present the spatial gradients across the array (approximated by finite differences), which are necessary for deriving rotation rates. The stations used are indicated by black dots and those that are excluded by grey dots. In Figure 14, $\Delta v_{x}/\Delta y$ is shown in blue and $\Delta v_{y}/\Delta x$ in green. The blue and the green curves are both different for any other two neighboring stations. It means that the corresponding gradients are not uniform across the array and we cannot expect to obtain a satisfactory approximation of the torsion rate by the ADR method. Figure 15 shows the spatial gradients used to derive tilt rates: $\Delta v_{z}/\Delta x$ in blue and $\Delta v_{z}/\Delta y$ in green. We see that they are much more similar to each other than in Figure 14, especially $\Delta v_{z}/\Delta y$ (green curves), i.e., the best ADR estimate (but still far from being accurate) can be expected for $\Omega_{x}$ (axis in the N-S direction).

### 3.2. Comparison of Rotational Seismograms and Discussion

The active experiment that is described here took place at a distance of only several wavelengths from the blast site. Consequently, the seismograms cannot be easily separated to individual wave phases and the wavefield may also be affected by near-field effects. That is why the seismogram shapes are different from what is usually observed when measuring seismic rotations of local micro-earthquakes. Nevertheless, our comparative study relies on comparing the seismograms obtained by the three independent methods and not on analyzing the seismogram shapes themselves. On the other hand, the proximity to the source contradicts the ADR assumption of uniform spatial gradients and the question is to what extent. Note that, to obtain a better approximation of near-source spatial gradients, one could consider a higher-order approximation in Equation (3) and modify the ADR method accordingly. However, that would require more sensors measuring true ground motion (i.e., not suffering from various instrument-related problems) in the array than the number of the Le-3DLite instruments that the authors had in their disposal.

The results of the experiment can be slightly affected by temperature and atmospheric pressure. In the case of Rotaphone and ADR, those effects are not so important, because we measure the differences between sensors. Provided the effects are the same or similar for all sensors, the spatial gradients are insensitive to atmospheric conditions. On the contrary, the R-1 sensitivity to temperature may be an issue [22].

**Figure 16:**
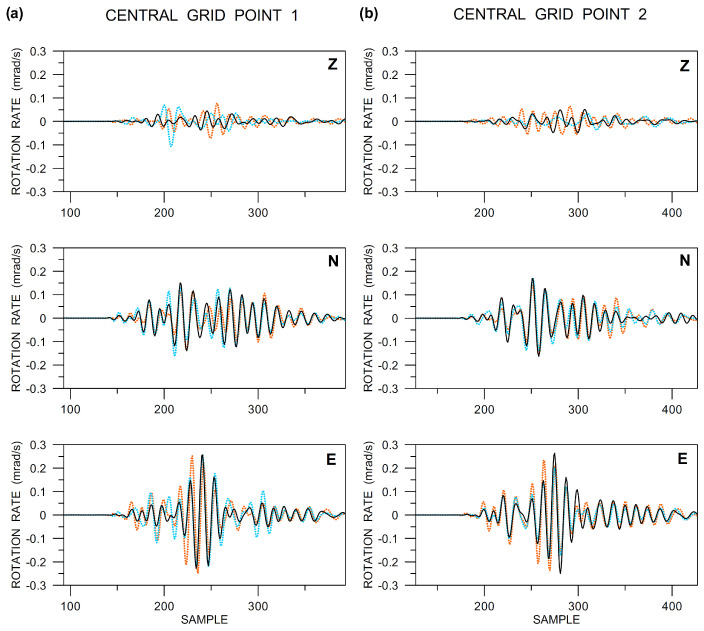
Comparison of the ADR (dotted orange), Rotaphone (solid black), and R-1 rotation rate components (vertical Z, North N, and East E) at the central grid points 1 (**a**) and 2 (**b**). The data are band-pass filtered between 6 and 20 Hz and instrument-corrected. The sampling frequency was 250 Hz. The Rotaphone and ADR amplitudes are real. The amplitudes of the R-1 records are multiplied by the following normalizing factors: 10 (Z), 5 (N), 2 (E) in part (**a**), and 1 (Z), 1.8 (N), 1 (E) in part (**b**).

In Figure 16, there are the rotational components at the two central grid points, as obtained from the ADR method (orange) and recorded by the R-1 sensors (light blue) and Rotaphones (black). Concerning the Rotaphone data, they show a significant similarity when comparing the two central points (2 m apart from each other), although the measurements at the two points were totally independent and different Rotaphone instruments were involved. On the other hand, studying details in the seismograms, certain differences between the two central points can be seen, which indicates that, in the given frequency window (up to 20 Hz), the spatial gradients may somewhat change even over such a small distance. This issue may be elucidated employing the other two methods. First, let us compare the ADR and Rotaphone results. Except for the torsion rate (Figure 16, top traces), the overall agreement is relatively good when taking into account that the records contain frequencies up to 20 Hz. In determining the torsion rate, the ADR method obviously fails, as it was explained when discussing Figure 14. On the contrary, the match is very good for the *x*-axis (N-axis) rotation rate (the middle traces in the figure). Remind that, according to our conclusions concerning $\Delta v_{z}/\Delta x$, drawn from Figure 15, the ADR is expected to work relatively well in this case. The ADR vs. Rotaphone data agreement is somewhat worse for the E-axis tilt, but still not being so bad, especially at times around the maximum amplitude and after. Figure 16, bottom, shows that the largest differences between the ADR and Rotaphone results are in those segments of the seismograms where the Rotaphone records at points 1 and 2 differ the most from each other. This could be explained in two ways: either the Rotaphone data are not correct in these parts of the seismograms, or the rotation rates really changed from point 1 to point 2, which the ADR method cannot ‘see’, as it averages the results over the array. A surprisingly good agreement between the Rotaphone E-axis rotation rate and that provided by the R-1 sensors at point 2 supports the second hypothesis. Regarding the R-1 records in Figure 16, their amplitudes are normalized to the maximum value from the other two methods (whose amplitudes are real). The reason is that we had found amplitude deviations from the specifications that were provided by the manufacturer during our laboratory tests preceding the quarry-blast experiment. Similar findings are also reported by other authors [21]. When comparing the waveforms, the R-1 records match the records from the other two methods in the N-axis component well, especially at point 2. The R-1 *z*-component does not seem to be reliable when taking into account the totally different records at the two central points (in contrast to the other two methods). Regarding the E-component at point 1, the R-1 waveform only roughly matches the other two waveforms and certain discrepancies are clearly seen. To summarize, the R-1 *z*-component seems to be wrong at both of the central points. At point 2, the R-1 tilt waveforms agree very well with those obtained by Rotaphone and even by ADR, where it is applicable (the N-component). At point 1, the match is considerably worse. There is one possible explanation for this relative disagreement. The R-1 sensors require several-minute ‘calm-down’ time until they get to a stabilized state after sensing a large motion, as mentioned in Section 2.1. The blast used here as an example followed only five minutes after another blast at approximately the same distance. Perhaps the time delay between the blasts was not long enough, especially for the R-1 sensor situated at point 1 to get ready for further measurement.

**Figure 17:**
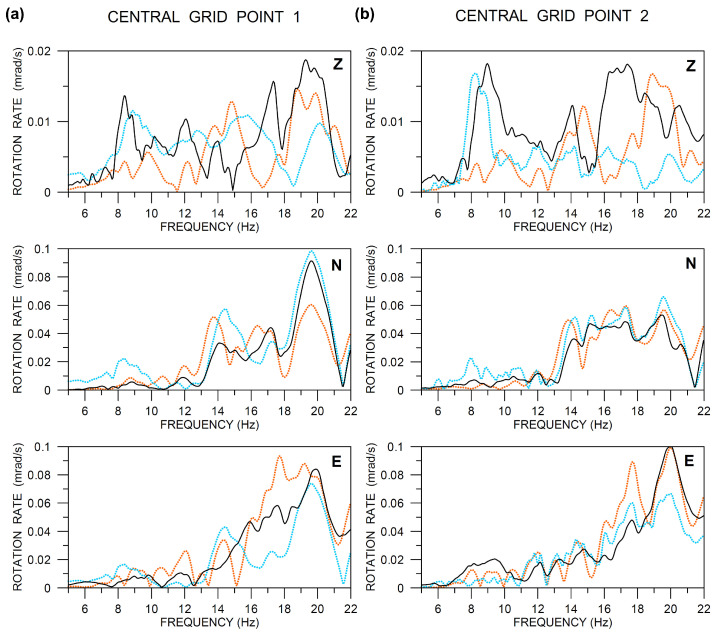
Comparison of the ADR (dotted orange), Rotaphone (solid black), and R-1 amplitude spectra (moduli) of three rotation rate components (vertical Z, North N, and East E) at the central grid points 1 (**a**) and 2 (**b**). Data are band-pass filtered between 6 and 20 Hz and instrument-corrected. The sampling frequency was 250 Hz. The Rotaphone and ADR amplitudes are real. The amplitudes of the R-1 records are multiplied by the following normalizing factors (the same as in Figure 16): 10 (Z), 5 (N), 2 (E) in part (**a**), and 1 (Z), 1.8 (N), 1 (E) in part (**b**).

It is also useful to compare the rotational records in the spectral domain (Figure 17). It confirms some conclusions that were drawn from Figure 16, e.g., a better mutual fit for horizontal components (tilt rates) compared to the Z-axis rotation (torsion) rate, as well as allows new information to be retrieved. First, in contrast to what one perhaps might expect, there is no overall better fit at lower frequencies (say, in the range 6–10 Hz) as compared to the higher ones. Second, there is an interesting peak around 20 Hz. While it is clearly visible in all components of the Rotaphone data at central grid point 1 (Figure 17a), it is significant only in the E-component at central grid point 2 (Figure 17b). The feature explains differences in the Rotaphones waveforms between the two central points, clearly visible in the N- and Z-components of Figure 16, in particular.

As already mentioned, the waveforms that were obtained by the three methods match each other relatively well only in certain time intervals. The *z*-component (torsion) does not even display any good fit at all. Therefore, in this particular experiment, it is not possible to verify the rotational records against each other.

The verification of rotational records is problematic in general, especially at small distances from the source and at higher frequencies. For distant earthquakes (and a low-frequency range, by nature), it would be possible to use the well-known rotation-to-translation relations [39] derived under the assumption of a plane wave with a constant amplitude along the wavefront. In those relations, the rotation rate around the vertical axis is proportional (with the opposite sign) to transverse translational acceleration and the rotation rate around the transverse horizontal axis is proportional to vertical translational acceleration. The matching of waveforms of the relevant components, measured as totally independent quantities, may then be a good way to verify rotational records. However, in our case, we cannot neglect (1) wavefront curvature and (2) amplitude variations along the wavefront, both due to directional source radiation and inhomogeneity of the medium. Brokešová and Málek [14] presented rotation-to-translation relations for a proximal directional point source in a homogeneous medium, according to which the above mentioned rotation rates are equated not to the corresponding acceleration terms only, but to a superposition of acceleration and velocity terms. Medium inhomogeneities may even reinforce the velocity terms in the relations because of stronger amplitude variations. Nevertheless, based on our experience with real measurements, the acceleration terms are still often dominant in the rotation-to-translation relations, even at small source-receiver distances and the presence of the velocity terms is manifested by a slight change in the waveform and a small, but apparent, phase shift between the relevant rotation rate and acceleration components. In light of the above, it is worth inspecting how rotational waveforms match those of accelerations.

**Figure 18:**
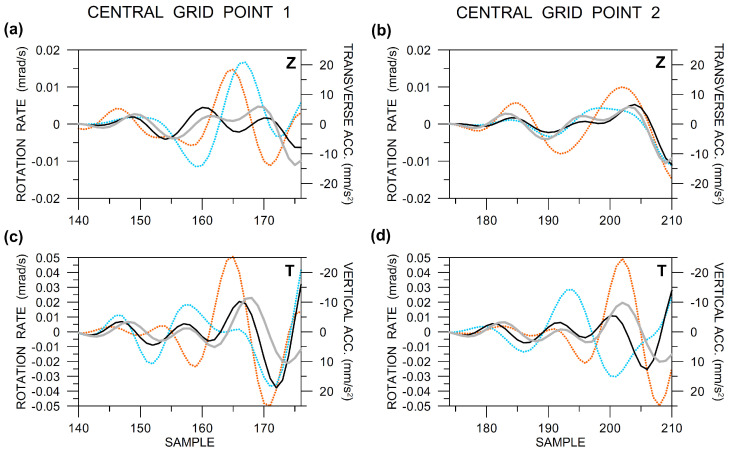
Comparison of the relevant acceleration and rotation rate components (vertical Z and transverse T) at the central grid points 1 (**a**,**c**) and 2 (**b**,**d**) in a short interval after the wave onset. Top (**a**,**b**): vertical rotation rate is compared to transverse acceleration, bottom (**c**,**d**): transverse tilt is compared to vertical acceleration. Colors: ADR—dotted orange, Rotaphone—solid black, R-1—light blue, acceleration—solid grey. Data are band-pass filtered between 6 and 20 Hz and instrument-corrected. The sampling frequency was 250 Hz. The amplitudes of the R-1 records are multiplied by the following normalizing factors: 10 (Z) in part (**a**), 1 (Z) in part (**b**), 5.3 (T) in part (**c**), and 1.8 (T) in part (**d**).

In order to perform the waveform matching, we need to know the transverse direction, which requires to know the exact azimuth the waves come. The problem is that in an inhomogeneous structure the true back azimuth may change relatively rapidly in time. Both Rotaphone and array measurements make it possible to determine the time-dependent actual back azimuth (e.g., the so-called zero-crossing-point method [40] would be particularly useful for the given small-aperture array) but that is beyond the scope of this study. Moreover, in inhomogeneous laterally varying structures, the wavefield is very complex, consisting of various multiply-reflected/scattered waves that are recorded at the same time in the latter phases of the seismograms. Therefore, here we focus on the beginning of the records, shortly after the wave onset, where there is a chance that the wavefield is not so complex and the waves come from the direction at least roughly corresponding to the geometrical back azimuth of the source. The acceleration-rotation rate waveform matching is shown in Figure 18. In its upper part, the figure provides a detail from the top of Figure 16, supplemented by the transverse acceleration (for the back azimuth of 320${}^{\circ }$) plotted in grey. In its bottom part, rotational records that are shown in the middle and bottom of Figure 16 are used to calculate the transverse tilt which is matched to the vertical acceleration. At both central points, the relevant acceleration components (grey) are best matched by the records that were made by the Rotaphones (black). In all cases, there are small phase shifts between the black and grey curves, which can be explained by the presence of velocity terms in the rotation-to-translation relations. The good fit may indicate good functionality of the Rotaphone-C design that is involved in the experiment and reliability of its rotational records.

## 4. Conclusions

The study presents the results of a measuring experiment able to compare recordings of seismic rotation rates obtained by three different methods: ADR, single-point measurements by Rotaphones, and single-point measurements by R-1 rotational sensors. At a distance of approximately 240 m from a medium-size quarry blast, we recorded short-period torsion rates of the order of magnitude of 10${}^{-5}$ and tilt rates of the order of magnitude of 10${}^{-4}$ rad/s. More specifically, the N-axis and E-axis tilt rates were approximately two times and three times, respectively, stronger in amplitude compared to the torsion rate along the *z*-axis. The three involved methods yielded rotational records matching each other only in parts, some of the records were only roughly similar to the others. The measure of agreement of the tilt rate records is much higher than that of the torsion rate records, especially when considering the whole waveforms. A comparison of the relevant rotation rate and acceleration components (theoretically similar according to rotation-to-translation relations) worked out the best for the Rotaphone records, which may be an indication of their reliability.

The experiment showed that, in the given frequency range, the rotation rate variation in space is far from insignificant. Apparent differences were registered between the two central points, 1 and 2, situated only 2 m apart. Non-negligible differences between the two central points can be seen also in the translational components. The differences, a consequence of various interfering waves, somewhat relativize the concept of collocated rotational and translational seismic measurements that were made by two instruments installed at a distance from each other, which is a common practice. Rotaphone records at points 1 and 2 showed differences in the waveforms, but the maximum amplitudes were very similar to each other. As the measurements at the two points were totally independent, the similarity in amplitudes may be considered as another indirect indication of a good functionality of the instrument.

The differences in rotation rates between the two central points also imply that the applicability of the ADR method is very doubtful, as its basic assumption of spatial gradient uniformity is obviously violated. Moreover, when applying the ADR method, it is difficult to ensure the same instrument responses of all the involved seismographs in the array—a huddle test that is similar to the one that we performed is insufficient. Thus, the ADR method only appears applicable with the greatest care, particularly in a short-period range. Certain supplemental tests of the applicability of the method, whenever possible, are of utmost importance.

The R-1 instruments proved to differ notably in certain parameters compared to the manufacturer’s specifications. An important problem appeared to be the relatively long calm-down time during which the instruments must be in a quiet environment to be ready for measurement after registering higher amplitudes due to manipulation or previously recorded stronger ground motions. The required calm-down time may cause limited applicability of such instruments, e.g., in continual recording of earthquake swarms with stronger events shortly following one after the other, recording of short-term aftershock sequences, or in seismic exploration procedures employing shots with short time delays.

## Figures and Tables

**Table 1:** Manufacturer specifications of the equipment involved in the experiment (only parameters relevant for the study): R-1 (top, manufacturer specification only), Rotaphone-C (middle, derived from SM-6 manufacturer specifications, A/D converter parameters and laboratory testing), and Lennartz LE-3DLite (bottom, derived from manufacturer specifications and A/D converter parameters).

Sensor		Translational Velocity	Rotation Rate
R-1	resolution	-	120 nrad/s
	hard-clip level	-	50 mrad/s
	frequency range	-	0.05–20 Hz
	temperature range	-	−15–+55 ${}^{\circ }$C
Rotaphone-C	resolution	0.647 nm/s	2.16 nrad/s
	hard-clip level	86 mm/s	287 mrad/s
	frequency range	2–60 Hz	
	temperature range	−20–+40 ${}^{\circ }$C	
Le-3DLite	resolution	3.125 nm/s	-
	hard-clip level	50 mm/s	-
	frequency range	1–80 Hz	-
	temperature range	−20–+65 ${}^{\circ }$C	-
